# Evolutionary reversal of physical dormancy to nondormancy: evidence from comparative seed morphoanatomy of *Argyreia* species (Convolvulaceae)

**DOI:** 10.1093/aobpla/plae033

**Published:** 2024-05-30

**Authors:** D M Nethani H Gunadasa, K M G Gehan Jayasuriya, Jerry M Baskin, Carol C Baskin

**Affiliations:** Department of Botany, University of Peradeniya, Peradeniya, KY 20400, Sri Lanka; Postgraduate Institute of Science, University of Peradeniya, Peradeniya, KY 20400, Sri Lanka; Department of Botany, University of Peradeniya, Peradeniya, KY 20400, Sri Lanka; Postgraduate Institute of Science, University of Peradeniya, Peradeniya, KY 20400, Sri Lanka; Department of Biology, University of Kentucky, 101, T.H. Morgan Building, Huguelet Drive, Lexington, KY 40508-0225, USA; Department of Biology, University of Kentucky, 101, T.H. Morgan Building, Huguelet Drive, Lexington, KY 40508-0225, USA; Department of Plant and Soil Sciences, University of Kentucky, 105, Plant Sciences Building, Lexington, KY 40546-0312, USA

**Keywords:** Convolvulaceae, desiccation tolerant, dormancy reversal, nondormancy, physical dormancy, seed anatomy, water gap

## Abstract

*Argyreia* is the most recently evolved genus in the Convolvulaceae, and available information suggests that most species in this family produce seeds with physical dormancy (PY). Our aim was to understand the evolution of seed dormancy in this family via an investigation of dormancy, storage behaviour, morphology and anatomy of seeds of five *Argyreia* species from Sri Lanka. Imbibition, germination and dye tracking of fresh intact and manually scarified seeds were studied. Scanning electron micrographs and hand sections of the hilar area and the seed coat away from the hilar area were compared. Scarified and intact seeds of *A. kleiniana*, *A. hirsuta* and *A. zeylanica* imbibed water and germinated to a high percentage, but only scarified seeds of *A. nervosa* and *A. osyrensis* did so. Thus, seeds of the three former species are non-dormant (ND), while those of the latter two have physical dormancy (PY); this result was confirmed by dye-tracking experiments. Since >90% of *A. kleiniana, A. hirsuta* and *A. zeylanica* seeds survived desiccation to 10% moisture content (MC) and >90% of *A. nervosa* and *A. osyrensis* seeds with a dispersal MC of ~12% were viable, seeds of the five species were desiccation-tolerant. *A. nervosa* and *A. osyrensis* have a wide geographical distribution and PY, while *A. kleiniana*, *A. hirsuta* and *A. zeylanica* have a restricted distribution and ND. Although seeds of *A. kleiniana* are ND, their seed coat anatomy is similar to that of *A. osyrensis* with PY. These observations suggest that the ND of *A. kleiniana*, *A. hirsuta* and *A. zeylanica* seeds is the result of an evolutionary reversal from PY and that ND may be an adaptation of these species to the environmental conditions of their wet aseasonal habitats.

## Teaser

In *Argyreia* , the most recently split-off genus of the Convolvulaceae, some species produce seeds with physical dormancy, and some produce non-dormant seeds. Species with a wide distribution have seeds with physical dormancy, while those with a restricted distribution have seeds with no dormancy. Moreover, species that split-off early have seeds with physical dormancy. Physical dormancy is the ancestral dormancy class of the genus, since its sister groups only contain species that produce physically dormant seeds. Seeds of the species with non-dormant seeds have remnants of anatomical and morphological structures of seeds with physical dormancy. The evidence suggests that non-dormant seeds are an adaptation and reversal of physical dormancy in *Argyreia* , and species with non-dormant seeds split-off recently and have a restricted distribution.

## Introduction

Physical dormancy (PY) in seeds, is caused by seed/fruit coat water impermeability (Rolston 1978; Baskin *et al.* 2000), and it has been identified in 18 angiosperm families (Mahadevan and Jayasuriya 2013; Baskin and Baskin 2014). Convolvulaceae is the only family with species producing physically dormant seeds in the taxonomically most recently evolved Asterid clade (Baskin *et al.* 2000). However, the Convolvulaceae not only includes species that produce seeds with PY but also those that produce seeds with combinational dormancy (i.e. physical + physiological dormancy) and with no dormancy (Jayasuriya *et al.* 2008).

Jayasuriya *et al.* (2008) have determined the kind of dormancy, germination requirements and storage behaviour of 46 species representing all 11 tribes of the subfamily Convolvuloideae (sensu Stefanovic *et al*. 2002). They found PY and combinational (PY + PD) dormancy and non-dormancy within the family. Further, orthodox and recalcitrant storage behaviour types were present within the family. Orthodox seeds are desiccation tolerant and have high storability, while recalcitrant seeds are desiccation sensitive and have low storability (Hong and Ellis 1996). Further, Jayasuriya *et al.* (2009) investigated the seed coat and ‘water gap’ morphology and anatomy of these species and described the evolutionary trends in seed dormancy within the subfamily Convolvuloideae. They showed that species in tribe Erycibeae, which has a tropical rainforest origin, have non-dormant (ND) recalcitrant seeds, while species in tribes Chardiochlaymeae and Cuscuteae in seasonal tropical climates have orthodox seeds with PY. Further, although most species in tribe Cresseae have seeds with PY, some members of Cresseae growing in wet aseasonal tropical climates have ND seeds, e.g. *Bonamia menziesii* (Jayasuriya *et al.* 2009). Moreover, members of the tribe Maripeae (with a wet tropical distribution) have ND seeds, while most members of sister tribes [Jacquemontieae, Cresseae and Dichondreae (Stefanovic *et al.* 2003)] have seeds with PY (Jayasuriya *et al.* 2009).

Ipomoeeae, the most recently evolved tribe in the Convolvulaceae, has a high number of species with a wide distribution in tropical and temperate habitats (Stefanovic *et al*. 2003; Mabberley 2017). Despite this wide geographical distribution and a high number of species, all species in Ipomoeeae studied thus far have seeds with PY (Baskin and Baskin 2014). Gupta (2003) suggested that *Argyreia nervosa* seeds might have PY or ND. Stefanovic *et al.* (2003) included *Argyreia*, *Binkworthia, Lepistemon, Lepistermonopsis, Paralepistemon, Rivea, Stictocardia* and *Turbina* in tribe Ipomoeeae based on molecular data. However, Austin (1973, 1998) and Wilkin (1999) included these genera in a separate tribe (Argyreieae) based on morphological, anatomical and physiological data. Moreover, Stafnovic *et al.* (2002) identified *Argyreia* and *Rivea* as forming a monophyletic group, but these two genera are well nested within the cluster formed by other Ipomoeeae genera. Seed dormancy and anatomy of tribe Argyreieae (*sensu*Austin 1998) have not been studied in detail; this tribe was not included in the studies by Jayasuriya *et al*. (2008, 2009) on seed dormancy/anatomy of the Convolvulaceae. Thus, the possibility that tribe Argyreieae may contain some species with ND seeds and others with physically dormant seeds makes this an important tribe with which to further explore the evolutionary relationship between ND and PY in the Convolvulaceae.

To contribute or our understanding of the evolution of seed dormancy in the family Convolvulaceae, we investigated the dormancy, morphology and anatomy of seeds of five *Argyreia* species, which represent the most recently split-off sub-tribe of the family: *Argyreia nervosa* (Burm. f.) Bojer, *Argyreia kleiniana* (Schult.) Raizada, *Argyreia hirsuta* Wight & Arn.. *Argyreia osyrensis* (Roth) Choisy and *A. zeylanica* (Gaertn.) Voigt. *Argyreia* has a paleotropical origin and distribution (Eserman *et al*. 2014), and it occurs in South and Southeast Asia and Madagascar (IPNI 2022). We hypothesized that seeds of *Argyreia* species occurring in aseasonal habitats such as tropical rainforests have ND seeds, while sister species growing in seasonal habitats have seeds with PY.

## Methods and Materials

### Study species

All five study species of *Argyreia* are perennial lianas. *A. nervosa* has a wide native distribution in Asam, Nepal, Bangladesh, India and Myanmar, and it has been introduced and naturalized in many other Asian (including Sri Lanka) and African countries (IPNI 2022). This species occurs in rainforests, open woodlands, waste ground, riverbanks and roadsides of wet habitats up to 900 m a.s.l. (Padhi *et al*. 2013). *A. osyrensis* occurs in Sri Lanka and in southern India to Burma, and it grows in disturbed sites in the dry zone (Austin, 1980). *A. kleiniana* is native to Sri Lanka and the Western Ghats of India, and it is found in rainforests, open woodlands, waste grounds and roadsides of lowland wet habitats (Austin 1980). *A. hirsuta* is found only in the Nilagiri Mountains of India and the hill country of Sri Lanka. Its habitats are the same as those of *A. kleiniana,* but the species occurs only above 600 m a.s.l. (Austin 1980). *A. zeylanica* is found only in southern India and northeastern Sri Lanka in the dry zone. It occurs in disturbed sites similar to those of other species.

### Fruit collection and seed extraction

Cleaned non-treated seeds of *A. nervosa* were purchased from Nurserylive, Magarpatha, Maharashtra, India. Fruits of *A. osyrensis, A. kleiniana*, *A. hirtusa* and *A. zeylanica* were collected from at least five lianas in Bowatenna (dry zone), University of Peradeniya, Peradeniya (Wet zone), Knuckles conservation area (Intermediate zone) and Rambaken-Oya nature reserve, Ampara, Sri Lanka, respectively, during their peak dispersal time (Table 1). All the fruits were collected from plants (and not from the ground) and care was taken to collect only (yellow-coloured soft mature fleshy fruits at dispersal maturity vs. green-coloured hard non-matured fruits of *A. kleiniana, A. hirtusa* and *A. zeylanica* and red-coloured dry mature fruits vs. green-coloured freshy nonmature fruits of *A. osyrensis*). Collected fruits were placed in brown paper bags and transported to the seed biology laboratory at the University of Peradeniya. Fleshy fruits of *A. kleiniana*, *A. hirtusa* and *A. zeylanica* were immersed in water to separate seeds. Seeds were washed thoroughly to remove debris and air dried for 3 hr to remove surface water, after which they were stored in plastic bottles until used for laboratory experiments. Seeds of *A. osyrensis* were removed from the dry fruits by hand. Laboratory experiments were initiated for the four species collected from the wild within 3 days after collection, while those for *A. nervosa* were initiated within 3 days from the reception of the samples. Moreover, seeds of *A. nervosa* were collected about 1 month prior to shipping, and shipping the material from India to Sri Lanka took 8 days. *A. nervosa* seeds were stored in plastic bottles at ambient room temperature conditions (~ 32 ^o^C, 55% RH).

**Table 1.** Seed collection site and time, seed dispersal time and habitat conditions as given in Austin (1980).

Species	Seed collection		Rainy season of the habitat (Survey Department of Sri Lanka, 2012)
	Site	Time	
*Argyreia nervosa*	Seeds were purchased		
*A. osyrensis*	From a secondary forest in Bowatenna	June, 2022	October to February
*A. kleiniana*	Roadside in Peradeniya	May, 2022	April to December
*A. hirsuta*	Roadside in Riverston, Knuckles forest reserve	October, 2022	April to December
*A. zeylanica*	Secondary forest in Rambakan oya, Amapara	November, 2022	October to February

### Seed dormancy and germination

Experiments were conducted to determine if seeds of the five study species had PY. If seeds have PY, manually scarified seeds imbibe water and germinate within a few days, while non-treated intact seeds do not. If nontreated intact seeds imbibe water and germinate within a few days, they are ND.

#### Seed germination.

 Four replicates of 15 non-treated intact seeds of *A. nervosa, A. osyresnsis* and *A. zeylanica* and 25 non-treated seeds each of *A. kleiniana,* and *A. hirtusa* were placed on tissue papers moistened with distilled water in 9-cm-diameter Petri dishes. The number of seeds used per replicate differed depending on the availability of seeds. Seeds were incubated at laboratory temperature (~25 ^o^C) and light (diffused sunlight and white fluorescent light during the daytime and complete darkness during the night [12 hr]) conditions. Seeds were observed for germination and signs of imbibition (seed coat rupture and swelling of seeds) at 2-day intervals for 30 days or until all seeds had germinated. Emergence of the radicle to a length of ~1 mm was the criterion for germination.

#### Imbibition.

 Ten manually scarified (with a single-edge razor blade) and 10 non-treated intact seeds each of *A. nervosa* were placed on tissue papers moistened with distilled water. Each seed was retrieved after 0, 2, 4, 6 and 24 hr of incubation and then at 1-day intervals for 10 days or until all seeds had germinated, blotted dry, reweighed and returned to the Petri dishes. The same experiment was conducted for *A. osyrensis* using 25 intact non-treated and 25 manually scarified seeds and for *A. kleiniana, A. hirtusa* and *A. zeylanica* using 15 intact non-treated seeds. Imbibition curves were constructed and compared.

#### Dye tracking of pathway of water entry into seeds.

Dye tracking was conducted to determine the potential pathway of water entry into the seeds of *A. kleiniana, A. hirsute, A. nervosa* and *A. osyrensis*. Five non-treated intact seeds each of *A. hirsuta A. kleiniana, A. nervosa* and *A. osyrensis* were immersed in a saturated methylene-blue solution. Seeds were retrieved after 1, 2, 4, 6 and 24 hr of immersion, cut into halves and observed under a dissecting microscope. The path of dye entry (or not) into the seed was stained blue. This experiment was not conducted for *A. zeylanica*.

### Seed moisture content

Moisture content of seeds was determined as soon as seeds were extracted from fruits i.e. as soon as seeds were brought to the seed biology laboratory. Thus, the measured seed moisture content of five study species except for *A. nervosa* can be considered as the moisture content of fresh seeds. Five replicates of three halved seeds each of *A. nervosa* and *A. osyrensis* and five replicates of 15 halved seeds each of *A. hirsuta*, *A. kleiniana* and *A. zeylanica* were weighed with a digital analytical balance to the nearest 0.001 g. Seeds were oven dried at 120 ^o^C for 3 hr and reweighed. SMC was calculated on a fresh mass basis using the following equation.


$$\begin{array}{lll} & Seed\;moisture\;\;content=\; & \frac{\left(Initial\;\;weight\;\text{-}\;\;oven\;\;\;dry\;\;weight\right)}{Initial\;\;weight}\;\times \;100\;\;\; \end{array}$$

### Effect of desiccation on seed viability and seed desiccation sensitivity

Experiments were conducted to determine the desiccation sensitivity/tolerance of seeds of the five study species. Seeds that can survive <10% seed moisture content (SMC) were categorized as desiccation tolerant, and those that lose viability when dried to <10% SMC were categorized as desiccation sensitive.

This experiment was conducted only for seeds of *A. hirsuta*, *A. kleiniana* and *A. zeylanica*. Five samples containing three replicates of 15 seeds (5 samples × 3 replicates × 15 seeds for each species) each of the species were weighed and placed in open Petri dishes in a desiccator. Seed samples were retrieved at 1-day intervals and reweighed. When seeds attained ~10, 20 and 30 % SMC, a sample (three replicates) was placed on tissue papers moistened with distilled water in Petri dishes and incubated at ambient laboratory temperature and light conditions as described above. Seeds were observed for germination at 1-day intervals until all seeds had germinated or rotted. Percentage viability of seeds was determined based on seed germination percentage.

### Seed morphology

Seeds of each of the study species were fixed to metal plates with double gum tape and sputter coated with gold/palladium mixture. Scanning electron micrographs of the hilum and bulge area were taken with a Zeiss EVO LS15 high-performance variable pressure environmental scanning electron microscope. This experiment was not conducted for *A. zeylanica*.

### Seed anatomy

Transverse sections of the seed coat at the hilum, bulge and seed coat away from the hilum of *A. osyrensis* and *A. kleiniana* were made using a single-edge razor blade. Sections were mounted on glass slides and observed and micrographed using an Olympus DP74 camera connected to the Olympus BX53 light microscope.

### Analysis of data

For all five species, germination data were analysed with the non-parametric Kruskal–Wallis test since the data did not follow a normal distribution. For *A. nervosa* and *A. osyrensis,* the final imbibition percentages of nontreated and scarified seeds were analysed separately with a Mann–Whitney test. Linear regressions were fitted separately for viability and moisture content of *A. kliniana*, *A. hirsuta* and *A. zeylanica* seeds to determine whether viability was reduced with decreasing moisture content.

## Results

### Seed dormancy and germination

#### Germination.

**Figure 1.**
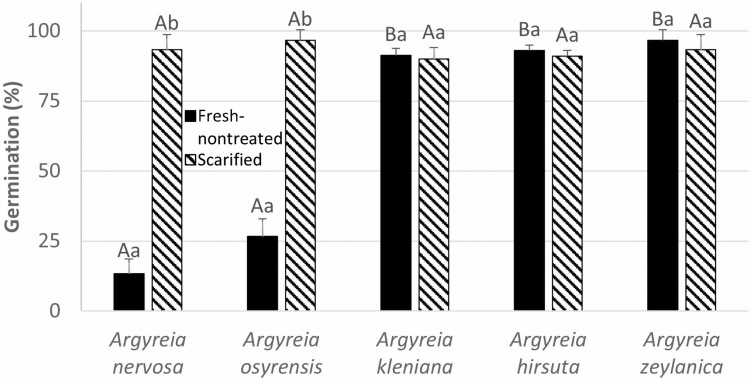
Germination percentage of fresh intact seeds of *Argyreia hirsuta*, *A. kleiniana A. nervosa*, *A. osyrensis* and *A. zeylanica* at ambient laboratory temperature (~25 ^o^C) and light/dark conditions. Different lowercase letters indicate significant differences between scarified and nonscarified seeds within the same species and different uppercase letters significant differences between species within the same treatment. Error bars are + 1 SE.

 Fresh intact seeds of *A. nervosa* and *A. osyrensis* germinated to 13.3% and 26.7%, respectively, within 30 days, while those of *A. kleiniana*, *A. hirsuta* and *A. zeylanica* germinated to >90% (Fig. 1). Germination percentages of the five study species differed significantly ((*H*[*χ*^2^] = 25.8, *P* = 0.001). Manually scarified seeds of *A. nervosa* and *A. osyrensis* germinated to 93.3 ± 5.4 and 96.7 ± 3.8%, respectively, while the few remaining seeds rotted. Non-scarified seeds of *A. nervosa* and *A. osyrensis* that did not germinate within 30 days did not show any sign of imbibition, while scarified seeds and germinated nonscarified seeds increased in size and their seed coats were ruptured before germination.

#### Imbibition.

**Figure 2.**
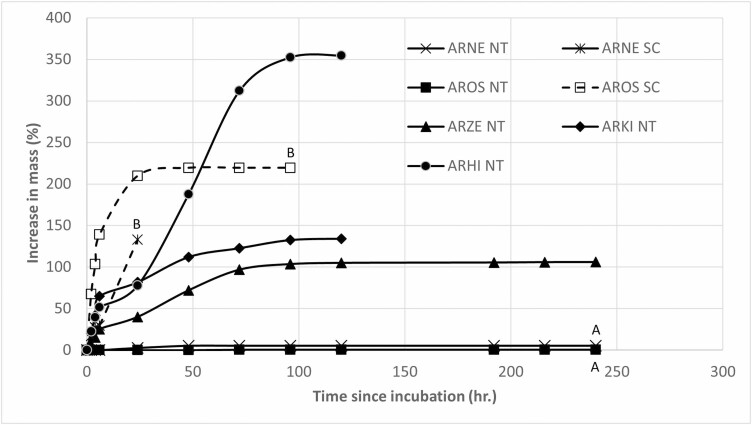
Mass increase (percentage) of intact and scarified seeds of *Argyreia nervosa* and *A. osyrensis* and of intact seeds of *A. hirsuta*, *A. kleiniana* and *A. zeylanica* during imbibition at ambient laboratory temperature (~25 ^o^C) and light/dark conditions. ARHI NT, *Argyreia hirsuta* nontreated intact; ARKL NT, *Argyreia kleiniana* nontreated intact; ARNE NT, *Argyreia nervosa* nontreated intact; ARNE SC, *Argyreia nervosa* scarified; AROS NT, *Argyreia osyrensis* nontreated intact; AROS SC, *Argyreia osyrensis* scarified; and ARZE NT, *A. zeylanica* nontreated intact. Different uppercase letters indicate significant differences between the final mass increase of scarified vs. intact seeds of the same species (AROS NT vs. AROS SC and ARNE NT vs. ARNE SC).

Increase in mass of intact nontreated seeds of *A. kleiniana*, *A. hirsuta* and *A. zeylanica* seeds was > 100 % (Fig. 2), while that of intact nontreated seeds of *A. nervosa* and *A. osyrensis* was <10%. In contrast, scarified seeds of these two species had a mass increase of >100%. Mass increase percentages of intact nontreated seeds of *A. nervosa* (*U* = 3.74, *P* < 0.001) and *A. osyrensis* (*U* = 4.72, *P* < 0.001) were significantly lower than that of scarified seeds.

#### Dye tracking of pathway of water entry into seeds.

**Figure 3.**
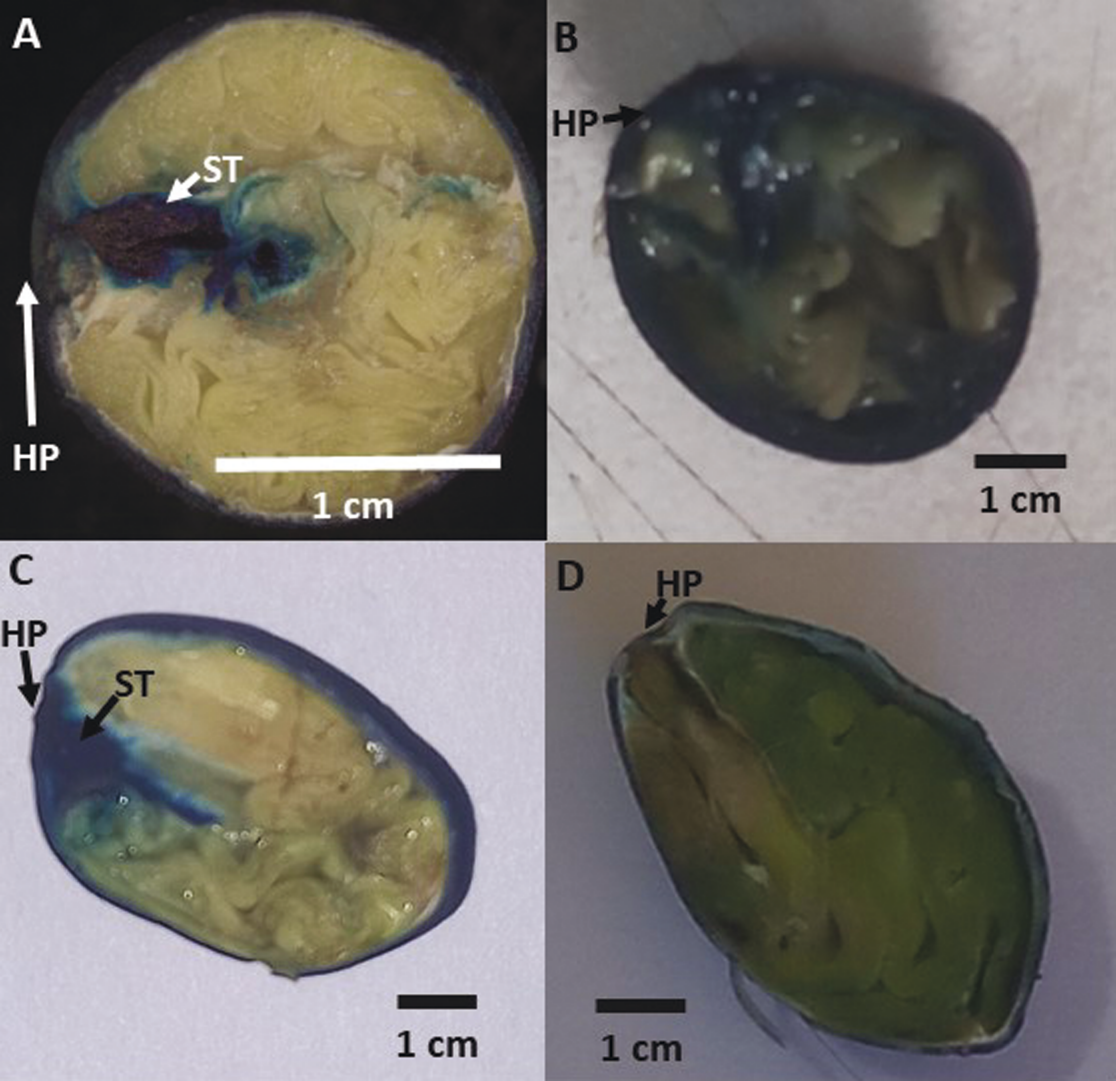
Seeds of *A. kleiniana* after 2 hr (A) and after 24 hr (B) and of *A. hirsuta* after 2 hr (C) and after 24 hr (D) of methylene blue staining. HP, hilar pad; ST, methylene blue dark staining.

No staining was observed inside any of the seeds after 1 hr of soaking in methylene blue. However, after 2 hr blue staining was observed below the hilum pad in the seeds of *A. kleiniana* (Fig. 3A) and *A. hirsuta* (Fig. 3C). After 4 hr, the whole inside of *A. kleiniana* (Fig. 3B) and *A. hirsuta* (Fig. 3 D) seeds was stained. Even after 24 hr, no staining was observed inside the seeds of *A. nervosa* or *A. osyrensis* (data not shown).

### Seed moisture content

*A. hirsuta* had the highest fresh seed mass and *A. osyrensis* the lowest (Table 2). The same trend was observed in seed dry mass. *A. nervosa* and *A. osyrensis* had an SMC < 15%, and the other three species had an SMC > 30%, at dispersal. SMC of the five study species differed significantly (*H*[*χ*^2^] = 35.8, *P* < 0.001).

**Table 2.** Fresh and dry mass and seed moisture content of the five study species.

Study species	Mean (± SE) fresh mass per seed (g)	Mean (± SE) dry mass per seed (g)	Mean (± SE) seed moisture content (%)
*Argyreia nervosa*	0.10 ± 0.02	0.091 ± 0.02	13.1 ± 1.4^a^
*A. osyrensis*	0.015 ± 0.001	0.012 ± 0.001	12.7 ± 0.7^a^
*A. kleiniana,*	0.14 ± 0.03	0.09 ± 0.02	32.1 ± 4.2^b^
*A. hirsuta*	0.45 ± 0.05	0.26 ± 0.04	42.0 ± 5.8^c^
*A. zeylanica*	0.32 ± 0.05	0.12 ± 0.03	64.1 ± 4.4^d^

Different lowercase letters indicate significant differences between species.

### Effect of drying on seed germination and seed desiccation sensitivity

**Figure 4.**
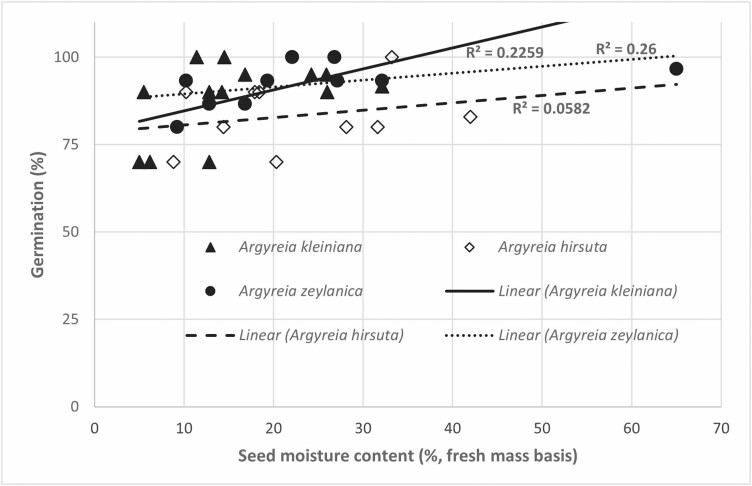
Viability of *Argyreia hirsuta, A. kleiniana* and *A. zeylanica* seeds dried in a silica gel desiccator to different seed moisture contents. Linear regression lines were fitted to show the trend in change of the viability.

Within about 3 days, SMC of *A. hirsuta*, *A. kleiniana* and *A. zeylanica* was <10%, and there were no significant differences in germination (viability) percentage for seeds dried to different seed moisture contents (*A. kleiniana*: *H*[*χ*^2^] = 4.06, *P* = 0.23, *A. hirsuta*; *H*[*χ*^2^] = 1.07, *P* = 0.57, *A. zeylanica*; *H*[*χ*^2^] = 3.98, *P* = 0.21) (Fig. 4). Although there were positive linear trends in germination, they did not differ significantly from a parallel line (*R*^2^ = 0.22, *P* = 0.10 [*A. kleiniana*], *R*^2^ = 0.06, *P* = 0.50 [*A. hirsuta*] and *R*^2^ = 0.26, *P* = 0.14 [*A. zeylanica*]).

### Seed morphology

**Figure 5.**
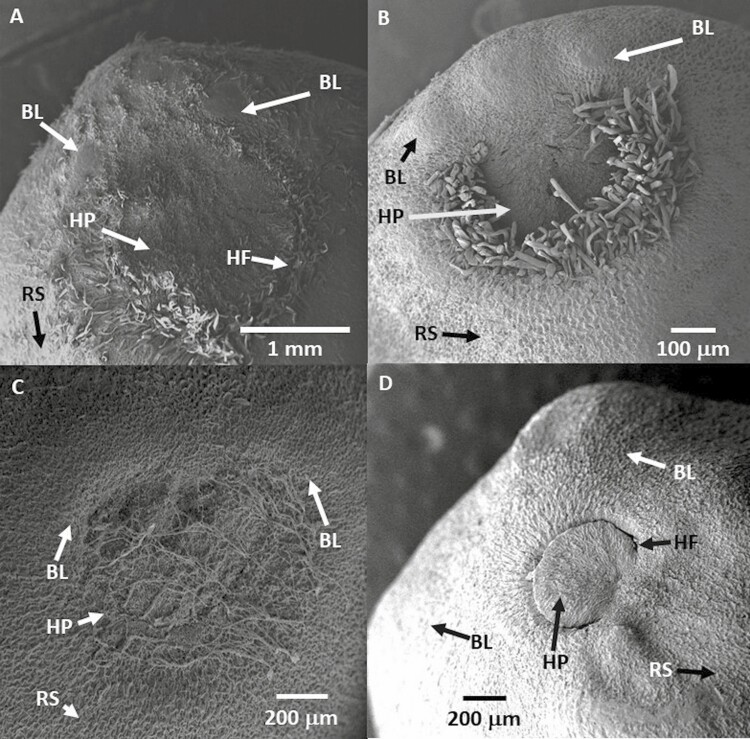
Electron micrographs of *Argyreia nervosa* (A), *A. osyrensis* (B) *A. kleiniana* (C) and *A. hirsuta* (D) seeds showing the hilum and bulge area. BL, bulge; HF, hilum fissure; HP, hilum pad; RS, seed coat away from hilum.

There were similarities as well as differences in the morphology of seeds of the five study species (Fig. 5). Seeds of the four species (*A. kleiniana, A. hirsuta, A. osyrensis* and *A. nervosa*) have a hilum pad and bulges [water gap in seeds of Convolvulaceae (Jayasuriya *et al.* 2009)]. The whole surface of *A. nervosa* seeds has trichomes (Fig. 5A), while trichomes were observed only on the hilum pad of *A. kleiniana* and around the hilum pad of the *A. osyrensis* seeds (Fig. 5C). The hilum pad of *A. kleiniana* has short trichomes, which normally were not visible to the naked eye. In contrast, trichomes on *A. nervosa* seeds are not densely arranged. They were visible to the naked eye and longer than those of *A. kleiniana*. *A. hirsuta* and *A. zeylanica* seeds did not have trichomes. Bulges on *A. osyrensis* seeds were more prominent than those on seeds of *A. kleiniana, A. hirsuta* and *A. nervosa*. On the other hand, *A. nervosa* seeds also have more prominent bulges than those of *A. kleiniana*, *A. hirsuta* and *A. zeylanica*. The hilum pad and the seed coat away from the hilar area of *A. nervosa, A. hirsuta* and *A. zeylanica* were separated by a clear hilum fissure, but there was no obvious hilum fissure on *A. kleiniana* or *A. osyrensis* seeds.

### Seed anatomy

**Figure 6.**
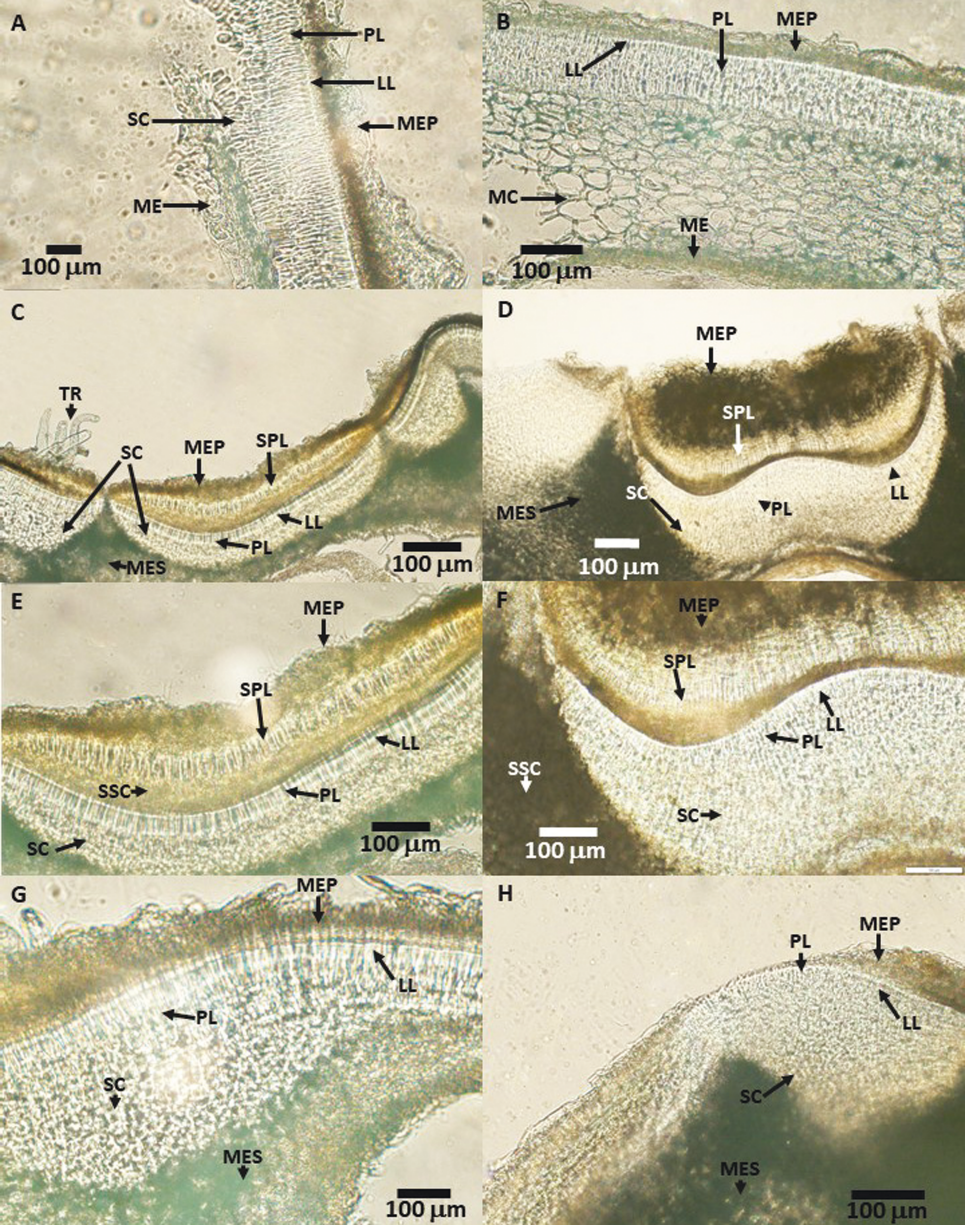
Micrographs of hand sections of *Argyreia osyrensis* (A, C, E, G) and *Argyreia kleiniana* (B, D, F, H) showing seed coat away from the hilum (A, B), hilum and hilum fissure (B, C), hilum (E, F) and the bulge (G, H) area. LL, light line; MC, mesophyll cell layer; ME, macerated cell layer below the seed coat; MEP, macerated cell layer above the seed coat; PL, palisade layer; SC, sclerenchyma cell layer; SPL, second palisade layer; SSC, second sclerenchyma layer.

The anatomy of the seed coat away from the hilum area of *A. osyrensis* and *A. kleiniana* seeds was similar to that of a typical water-impermeable seed coat. The outermost layer of the seed coat of both species consists of macerated cells (Fig. 6A and B). Below the macerated cells, both species have a palisade layer with a distinct light line followed by several layers of sclerenchyma cells. A slight difference in seed coat anatomy could be observed in this layer, i.e. *A. osyrensis* has 4–5 sclerenchyma layers, while *A. kleiniana* has only 2–3 sclerenchyma layers. Below the sclerenchyma layer, the *A. osyrensis* seed coat consists of a macerated cell layer, which is probably the remnants of the nucellus or the megagametophyte, and the *A. kleiniana* seed coat has a living mesophyll cell layer between the macerated cell and the sclerenchyma cell layers.

The anatomy of the hilar area of *A. osyrensis* and *A. kleiniana* is different from that of the seed coat away from the hilar area (Fig. 6C and D). The hilar pad of *A. osyrensis* is surrounded by a sub-hilar area, which is separated from the hilar pad by the hilar fissure. However, the sub-hilar area is not present on the micropylar side of the seed. The outermost layer of the sub-hilar area consists of trichomes and macerated cells and then of anatomical layers that are similar to those of the seed coat away from the hilar area. However, the sclerenchyma area consists of more layers than that of the seed coat away from the hilar area. The sub-hilar area of the *A. kleiniana* seed is not as conspicuous as it is in *A. osyrensis*.

The hilar pad of the seeds of *A. osyrensis* and *A. kleiniana* is very similar (Fig. 6C–F). The outermost layer of the hilar pad on seeds of both species consists of macerated cells. A palisade layer without a light line can be observed below the layer of macerated cells. A layer of sclerenchyma cells is below the palisade layer. The palisade layer with the light line, which is comparable to the palisade layer of the seed coat away from the hilar area, occurs below the sclerenchyma layer. A second sclerenchyma layer is present below the palisade layer with the light line. Below the second sclerenchyma cell layer is a mesophyll cell layer, which may be cells remaining from the nucellus.

Seeds of *A. osyrensis* and *A. kleiniana* have two bulges slightly above the hilar area. The seed coat of the bulges is thicker than that of the seed coat away from the hilum area (Fig. 6G, H). The thickness of the sclerenchyma layer in the bulges is responsible for the increased thickness of the seed coat on the bulges.

There are no clear differences between the two species in the anatomy of seed coat away from the hilum, hilum area and bulges.

## Discussion

Imbibition and germination experiments clearly showed that seeds of *A. kleiniana A. hirsuta* and *A. zeylanica* are ND, since >95% of intact non-treated seeds imbibed water and germinated. Since seeds of *A. nervosa* and *A. osyrensis* germinated <30% and manually scarified seeds imbibed significantly higher amounts of water than the nonscarified seeds, they have PY. On the other hand, >90% survival during the desiccation of seeds of *A. kleiniana*, *A. hirtusa* and *A. zeylanica,* and high viability (>90%) at the dispersal moisture content (~12%) of *A. nervosa* and *A. osyrensis* revealed that they were desiccation tolerant. Thus, seeds of *A. kleiniana*, *A. hirsuta* and *A. zeylanica* which have a restricted distribution in Southern India and Sri Lanka (Austin 1980) produce desiccation-tolerant ND seeds, while *A. nervosa* and *A. osyrensis* with a wide distribution in the dry zone of India and Sri Lanka (Austin 1980) produce desiccation-tolerant physically dormant seeds.

Seed dormancy and desiccation sensitivity/tolerance are two important seed traits that determine the timing of germination (Rubio de Casas *et al*. 2012), and thus they are highly influenced by the environmental conditions in the habitat of a plant species. *A. kleiniana* and *A. hirtusa* are distributed strictly in high-humidity habitats where they are exposed to high pathogenic and predatory pressures (Vazquez-Yanes and Orozco Segovia 1984). As an escape strategy from these pressures, seeds of species in humid habitats tend to germinate faster than those in dry habitats (Dalling *et al.* 2011, 2020). Thus, it may be advantageous for the ND seeds of *A. kleiniana* and *A. hirtusa* to germinate quickly under the humid conditions of their habitat. In contrast, ND seed-producing *A. zeylanica* occurs in the dry zone of southern India and northeastern Sri Lanka. Nevertheless, this species disperses seeds at the beginning of the wet season (December, personal observation by KMGGJ), and thus fast germination may allow them to escape the high pathogenicity during the wet season of the dry zone. *A. nervosa* occurs in a wide range of habitats including seasonally dry ones (Padhi *et al.* 2013), and *A. osyrensis* occurs mainly in dry zone habitats including dry mixed evergreen forest fringe and disturbed secondary scrub in the dry zone (Austin 1980). As such, PY of *A. nervosa* and *A. osyrensis* seeds may be an adaptation to their habitats that synchronizes germination to a time favourable for seedlings to develop, i.e. the wet season. These two species occur in rather dry habitats and probably do not germinate during intermittent rains in the dry season because the seeds are dormant. Notably, the natural dormancy-breaking cues for *A. nervosa* and *A. osyrensis* seeds have not been determined. On the other hand, physical dormancy is also considered an adaptation to defend against seed predators (Paulsen *et al*. 2013; but see Jayasuriy *et al*. 2015) and pathogens (Dalling *et al*. 2020).

*Argyreia* is among the most recently split-off genera in the Convolvulaceae, and *Argyreia* and *Revia* form a monophyletic group well nested within the tribe Ipomoeeae (Stafnovic *et al*. 2002). All *Ipomoea* species and all other studied species in tribe Ipomoeeae are reported to have seeds with PY. Thus, PY can be considered to be the ancestral dormancy state in the genus *Argyreia,* and ND in seeds of *A. kleiniana*, *A. hirsuta* and *A. zeylanica* can be considered to be a derived character. Therefore, the evolution of ND from PY within the clade Argyreiae could be a result of the movement of *Argyreia* from dry to wet aseasonal habitats. However, *Argyreia* seeds have retained their ability to tolerate desiccation, although recalcitrancy is considered to be an adaptive advantage in wet aseasonal habitats (Pammenter and Berjak 1999).

The evolution of ND from PY in the genus *Argyreia* also is supported by the comparative anatomy and morphology of the seeds of *A. kleiniana* (ND) and *A. osyrensis* (PY). Seeds of both species have similar morpho-anatomy, including a conspicuous hilum area and a bulge similar to those in seeds of other Ipomoeeae species. Although the bulge of the ND *A. kleiniana* seeds is functionally not active (as a water gap), it is morphologically and anatomically distinguished from the rest of the seed coat and is similar to the (probably) functional bulge of *A. osyrensis* seeds that is the water gap of Convolvulaceae seeds with PY (as identified by Jayasuriya *et al.* 2009). Furthermore, the seed coat away from the bulge and the hilum of *A. kleiniana* seeds has anatomy similar to that of seed coats of Convolvulaceae seeds with PY, including those of *A. osyrensis*. The seed coat of ND seeds of *A. kleiniana* contains a palisade layer with a light line, a specific feature of seeds with PY (see Baskin and Baskin 2022), which likely is a relic of ancestors whose seeds had PY.

Among the five species studied, *A. nervosa* and *A. osyrensis* produce seeds with PY as well as dry dehiscent fruits, while *A. kleiniana*, *A. hirsuta* and *A. zeylanica* produce ND seeds and fleshy indehiscent fruits. Dry dehiscent fruits of Convolvulaceae generally are explosively dispersed, and seeds may be dispersed (primary dispersal) for a relatively short distance away from the mother plant. It is advantageous for these seeds to be dormant because it gives them time to be secondarily dispersed (Athugala *et al.* 2021). On the other hand, fleshy fruits are adapted for dispersal by frugivores. Thus, the seeds potentially can be dispersed for long distances and to specific sites suitable for germination, e.g. aseasonal habitats, or in the wet season of seasonal habitats where germination and seedling establishment can occur successfully without delay. Further, PY cannot develop in seeds in fleshy fruits because they require maturation drying to a species-specific low moisture content of ~8–13% to do so (Qu *et al.* 2010; Baskin and Baskin 2014; Jaganathan 2016, 2022). We speculate that the evolution of ND from PY and of fleshy fruits from dry dehiscent fruits may have occurred simultaneously. The dye-tracking experiment showed that water uptake (imbibition) in both *A. kleiniana* and *A. hirsuta* seeds is mainly through the hilar fissure. Moreover, even if the *A. kleiniana* seeds were desiccated they absorbed water through the hilar fissure (personal observations by first author).

There are 10 *Argyreia* spp. in Sri Lanka, and except for *A. nervosa* all of them are native to this country (Austin, 1980). *A. nervosa* was introduced from India to Sri Lanka as well as to many other countries. Among the nine species native to Sri Lanka, *A. hancorniaefolia* and *A. thwaitesii* are endemic to Sri Lanka and four species, *A. elliptica, A. hirsuta, A. zeylanica* and *A. kleiniana,* are restricted to southern India and Sri Lanka. *A. nervosa A. osyrensis* and *A. laotica* have a wider geographical distribution, occurring in peninsular India, Sri Lanka and other neighbouring countries like Burma. According to the descriptions given by Austin (1980; Table 3), all of the *Argyreia* species, except *A. nervosa* and *A. osyrensis* have indehiscent fleshy fruits. Thus, we can speculate that these species also produce ND seeds. Fruits of *A. nervosa* and *A. osyrensis* are dry and dehiscent, and both species produce seeds with PY. According to our study and speculations made based on the fruit type, species with a wide distribution have PY seeds (except *A. laotica*), while species with a restricted distribution have ND seeds.

**Table 3.** Distribution, fruit type and identified/speculated seed dormancy class of *Argyreia* species in Sri Lanka and of the species included in the phytogenetic analysis in Convolvulaceae.

	Current name	Synonym	Native/exotic	Distribution	Ecology	Fruit type	Dormancy class
Species in Sri Lanka	*Argyreia kondaparthiensis*	*A. choisyana*	Native (Extinct from SL)	Sri Lanka, India	Dry zone	No record	Could not infer
	*Argyreia elliptica*		Native	Sothern India, Central Sri Lanka	Montane wet zone >2000 feet	Fruits fleshy, indehiscent	ND^*^
	*Argyreia hancorniaefolia*		Endemic	Sri Lanka	Submontane zone	Fruits fleshy, indehiscent	ND^*^
	*Argyreia hirsuta*		Native	Central Sri Lanka, Nilagiri mountains India	Submontane zone	Fruits baccate, fleshy	ND
	*Argyreia nervosa* ^##^ ^^^		Exotic	Assam and Bengal to Mysore, Sri Lanka (as naturalized exotic)	Open woodlands, roadsides, disturbed sites and waste areas	Dry, dehiscent	PY
	*Argyreia osyrensis* ^##^ ^^^		Native	Cambodia, Hainan, India, Laos, Myanmar, Sri Lanka, Sumatra, Thailand, Vietnam	Disturbed sites in the dry zone of Sri Lanka	Dry, dehiscent	PY
	*Argyreia zeylanica*	*A. pomacea*	Native	Kerala, India and Sri Lanka	Dry zone of Sri Lanka	Fruits baccate, fleshy	ND
	*Argyreia kleiniana*	*A. populifolia*	Native	Kerala, India and Sri Lanka	Wet and dry zones in Sri Lanka	Fruits baccate, fleshy	ND
	*Argyreia laotica* ^##^ ^^^	*A. splendens*	Native	India, Burma, Sri Lanka	Montane wet zone > 2000 feet	Fruits baccate, fleshy	ND^*^
	*Argyreia thwaitesii*		Endemic	Sri Lanka	Wet zone Lowland	Fruits baccate, fleshy	ND^*^
Species not in Sri Lanka	*Argyreia mollis* ^^^	*A. obtecta*		China, Cambodia, India (Andaman Islands), Indonesia, Laos, Malaysia, Myanmar, Thailand, Vietnam	Dense forests wet zone in intermediate elavations	Fruits red or orange berry	ND^*^
	*Argyreia capitiformis* ^^^			China, Cambodia, NE India, Indonesia, Laos, Malaysia, Myanmar, Thailand, Vietnam	Open waste ground in wet zone	Fruits berry	ND^*^

^*^Inferred from fruit type and PD was excluded as none of the experimental species had PD.

^##^Included in the phylogenetic analysis of Stefanovic *et al.* (2002).

^^^Species included in the phylogenetic analysis of Manos *et al.* (2001).

Information about Sri Lankan species is either from observations or from Austin (1980); information about the other two species is from Ke *et al.* (1995).

**Figure 7.**
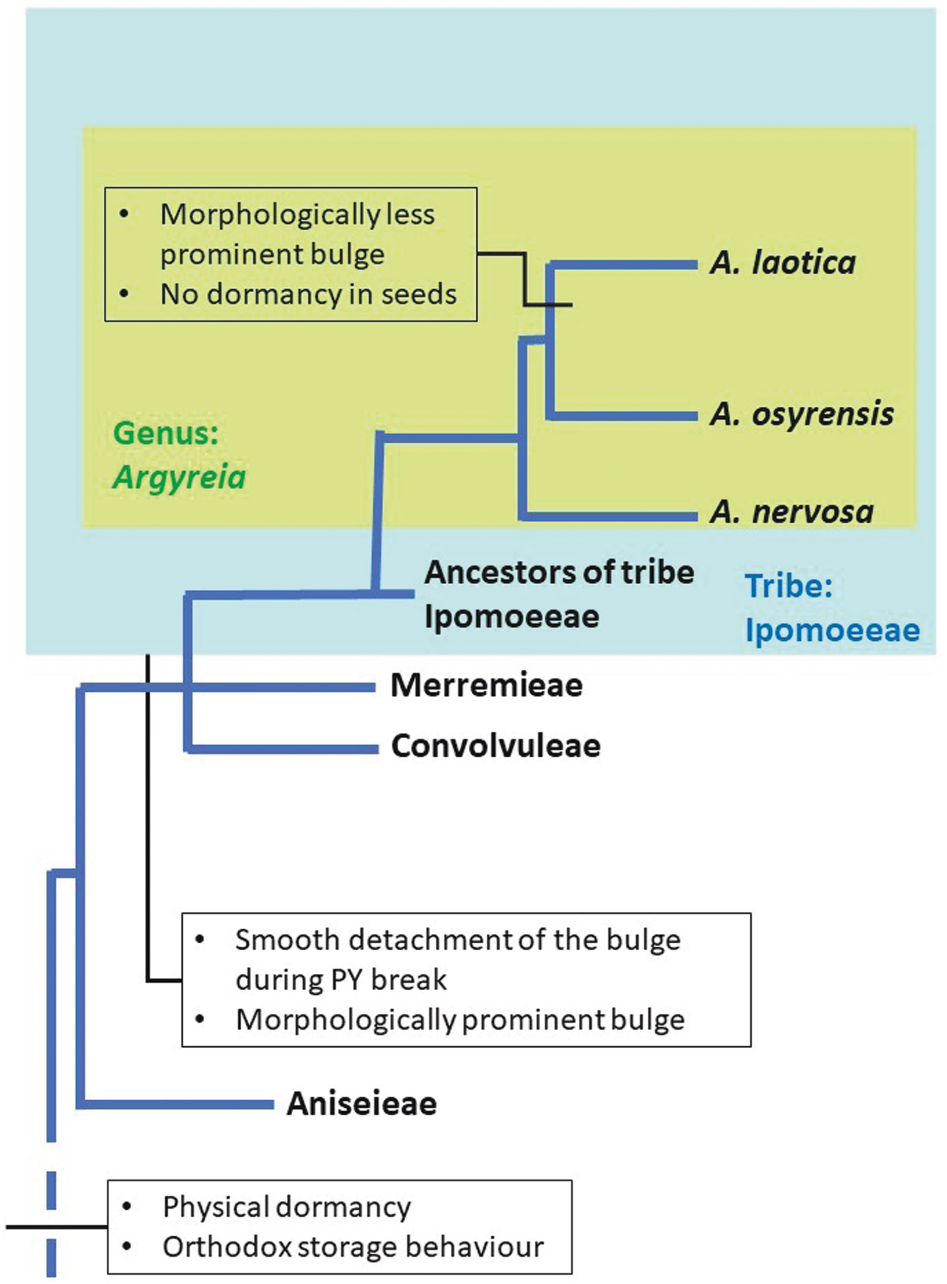
Proposed pathway for the evolution of seed dormancy in clade Convolvuloideae of the Convolvulaceae.

In the phylogenetic analysis by Stafanovic *et al*. (2002), *A. nervosa* is the out-group to *A. osyrensis* and *A. laotica* (syn. *A. splendens*), i.e. *A. laotica* and *A. osyrensis* have split-off more recently than *A. nervos*a. We speculate that the most recently split-off taxon, *A. laotica,* produces ND seeds (Table 3), while the out-group *A. nervosa* produces PY seeds. Moreover, based on the phylogenetic analysis by Manos *et al.* (2001), *A. nervosa* is the out-group to *A. osyrensis*, *A. laotica, A. mollis* and *A. capitiformis* (Fig. 7). According to Ke *et al*. (1995), fruits of *A. laotica*, *A. mollis* and *A. capitiformis* are berries, and thus we speculate that the seeds do not have PY. We suggest that ND is a derived character in seeds of these *Argyreia* species and that it is a reversal of PY to the most ancestral dormancy state of the family Convolvulaceae, i.e. ND in *Erycibe* and *Humbertia* as described by Jayasuriya *et al.* (2009).

Jayasuriya *et al.* (2009) suggested that the PY of species in Convolvulaceae evolved when the Convolvulaceae species dispersed to seasonal habitats like those in dry-tropical and temperate biomes. Further, these authors have shown that in several taxa PY of seeds has reverted back to ND as these species became redistributed in the aseasonal habitats like those in tropical rain forests. Tribe Maripeae in the clade Dicranostyloideae and *Bonamia menziesii* are examples of taxa in which PY has reverted back to ND, which is the most ancestral state of seed dormancy in the family. Our study showed that the same phenomenon has occurred in the most recently split-off genus (*Argyreia*) in the most recently split-off tribe (Ipomoeeae) in Convolvulaceae, where some *Argyreia* species that are distributed in aseasonal habitats (mainly the tropical wet habitats) have evolved ND seeds.

## Data Availability

The data and code underlying this article are available on GitHub, at; https://github.com/Gehanj/Gunadasa-et-al.-AoBPlants-supplimetory-data-
